# Instruction in Medical Ethics

**Published:** 1915-11-20

**Authors:** 


The Hospital

The Workers' Newspaper of Administrative Medicine and Institutional Life, Administration, National Insurance and Health.

No. 1536, Vol. LIX. SATURDAY, NOVEMBER 20, 1915. PRICE ONE PENNY.

### INSTRUCTION IN MEDICAL ETHICS.

The motion asking for an inquiry into the instruction of medical students in the special ethical relationships of professional life, and proposed by Dr. John C. McVail at the recent meeting of the General Medical Council, appears to have given rise to a good deal of alarm in the minds of some of those who took part in the discussion. The possible addition of new lectures to an already overburdened curriculum was one cause of dismay, while another was found in the fear that the public might regard such instruction as the formal and authoritative recognition of professional rules having a trade-union quality and motive. But as Dr. McVail disclaimed all intention in either of these directions—though the wording of his motion perhaps left his purpose open to misconstruction—these alarms may be dismissed as unnecessary, or, at all events, premature. What he wishes is to find out what is the present practice in relation to ethical instruction, and when this is known it will be for the Council to say whether more is or is not necessary. As the motion was carried, it will now rest with the Education Committee to prepare a report on the subject, and this report will certainly be anticipated with much interest.

Even those members of the Council who regarded Dr. McVail's proposal as unnecessary or dangerous admitted that in some fashion or another the medical student must be indoctrinated with the spirit which the profession cultivates and professes. In the view of some of the speakers this end is already attained by the influence of the tone and atmosphere of the medical school, whilst others place confidence largely in the personal relations established between teachers and students. No one will question the importance of these operations, and should they be wanting, the mere dictation of fine precepts would prove but a vain thing. Then, in some schools, most certainly, a greater or less measure of specific instruction is already attempted by one or more of the teachers, and it will be for the Education Committee to discover how far this extends, and whether there are or are not reasons for regarding it as beneficial. Should inquiry prove that these various methods, alone or in co-operation, are sufficient, there will be no need for any new proposal. At the worst, then, we shall have confident knowledge in a region where at present there is much uncertainty and vagueness. In our opinion, therefore, Dr. McVail's proposal was fully justified, and we congratulate him on its inception and success.

Without venturing to anticipate the result of the investigation now to be undertaken by the Education Committee, we have no hesitation in saying that students, in the course of the curriculum, ought to receive guidance relative to some of the anxious and difficult situations which arise in active professional life. It may be quite true, as one member of the Council remarked, that the principles of ethical behaviour are as old as the New Testament, and that conduct has a secure guidance in the golden rule. But the difficulties in certain circumstances of the application of these admirable precepts are proverbial, and youth ought to have the benefit of the experience which time has gathered. This can scarcely be attained by the sole announcement of pleasing maxims, however dignified these may be and however venerable their sanctions. To say that a man should "do to others as he himself would be done by" is an admirable article of general benevolence, but to apply it to a particular case, and especially a case in which there are varied and apparently conflicting interests, is often a puzzle in practical life. Few would adopt it towards a burglar, where, indeed, the position is a comparatively simple one. And, to take an individual professional instance, where is its guidance when the interests of a patient and those of a fellow practitioner appear to be in conflict? To leave the student without preparation for this and suchlike problems, or merely to feed him with vague phrases, is to subject him to the risk of blunders which will be creditable neither to himself nor to the profession to which he belongs. Even the best intentions, unless directed by knowledge, may pave the way to a most unpleasant place. We repeat that instruction of the order here indicated is certainly given in some of the medical schools, and for aught we know to the contrary it may be given in all. Whether there are any of the schools in which it is omitted and whether, if omitted, compensation is effected by other influence are points which the Education Committee is now in a position to determine.

There is one reason for the instruction of the student in professional ethics which appears to have escaped the recent discussion. It is the need for equipping the practitioner with the ability to defend professional procedure when this is challenged more or less widely by public opinion. Admittedly what is called "medical etiquette" is apt to be regarded with a suspicious eye by many laymen, and the reason for this is partly ignorance and partly misunderstanding. There is, of course, no "etiquette" which does not place the welfare of the patient as the chief and abiding interest. The public, however, will never understand this unless the individual member of the profession is prepared to show that each professional custom and practice is in harmony with this consideration. It is not enough to say that the rule for action is this or the other. What is necessary is to show that the rule has no narrow sectional or class motive, but that it is, on the contrary, adopted and practised in the public interest. It is not everyone to whom this comes "by nature," and hence the need for producing the informed practitioner by means of the education of the student.

